# Never Too Old? Occurrence of Medulloblastoma in the Elderly beyond the 70th Year of Life

**DOI:** 10.1155/2018/8639404

**Published:** 2018-06-07

**Authors:** Homajoun Maslehaty, Johannes Van de Nes, Sarah Teuber-Hanselmann, Christoph Moenninghoff, Ulrich Sure, Neriman Oezkan

**Affiliations:** ^1^Department of Neurosurgery, Nordstadt Hospital, Hannover, Germany; ^2^Institute of Neuropathology, Faculty of Medicine, University Duisburg-Essen, University Hospital Essen, Germany; ^3^Institute of Neuroradiology, Faculty of Medicine, University Duisburg-Essen, University Hospital Essen, Germany; ^4^Department of Neurosurgery, University Duisburg-Essen, University Hospital Essen, Germany

## Abstract

The occurrence of medulloblastoma (MB) in the elderly is an absolutely rare event. Concerning this issue we report on two MB patients beyond the 70th year of life. Two patients older than 70 years presented with a mass in the posterior fossa without evidence of a preexisting malignant tumor. After careful radiological work-up the suspected diagnosis was metastasis of an unknown primary tumor. Both patients underwent surgery and histopathological analysis revealed MB in both cases (classical MB and desmoplastic type). The two cases presented here represent also one classical MB and one additional desmoplastic MB. To our knowledge we report for the first time that there are different molecular subtypes of MB in the elderly patients that seem to be consistent with those subtypes mainly occurring in young adults. Unfortunately the patients died within one week after surgery due to respiratory insufficiency and an unclear cause. The presented cases show that MB can occur in the elderly. Although this constellation is absolutely rare, MB should be considered in the differential diagnosis, especially when a primary tumor is not known or detected.

## 1. Introduction

Medulloblastomas (MB) are small, round, blue cell, neuroectodermal tumors with neuronal differentiation in the cerebellum. These tumors usually occur in childhood. The incidence of MB in young adults is estimated to be less than 1% of all intracranial neoplasms [1, 2]. The mean age at time of diagnosis is estimated with 26 years [3]. The occurrence of MB in the elderly is an absolutely rare event. Concerning this issue we report on two MB patients beyond the 70th year of life.

## 2. Case Reports

**Figure 1:**
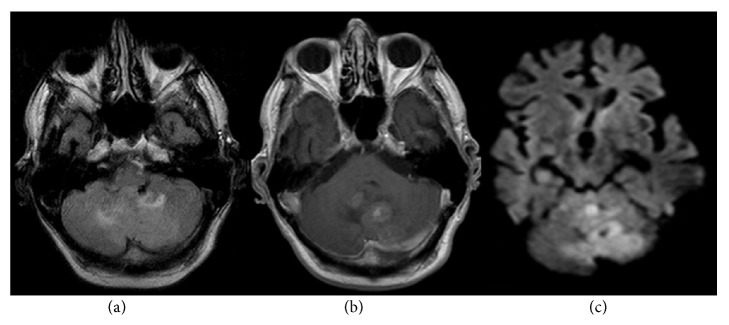
Transversal 1.5 Tesla FLAIR images (a) of a left cerebellar and vermal medulloblastoma show poor tumor delineation and inhomogeneous hyperintensity. Medulloblastomas normally appear hypointense relative to gray matter on T1w images with minimal to patchy contrast enhancement (b) and moderate restriction on diffusion weighted images (c).

**Figure 2:**
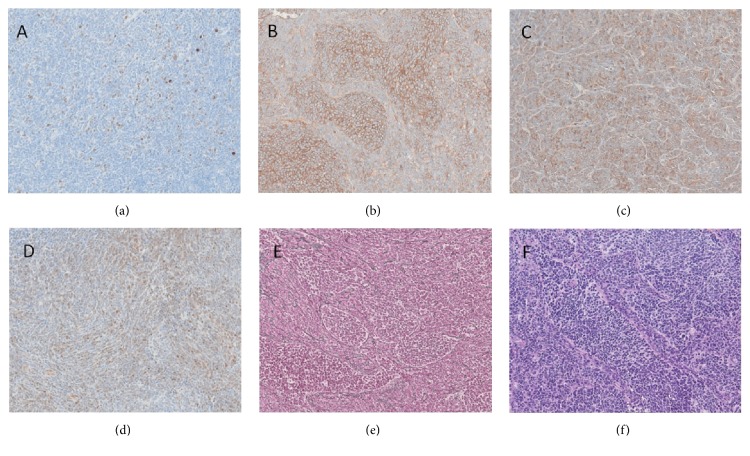
A small, round, blue cell, neuroectodermal tumor ((a), HE) showing a nodular architecture and a dense reticulin fiber network ((b), Gomori). Tumor cells showed nuclear expression of YAP1 (c) and cytoplasmic expression of GAB1 (d) while there was no nuclear expression of beta-catenin (e). Ki67-labelling reveals high proliferative activity (f).

Case 1 concerned a 71-year-old male presenting with gait difficulties and vertigo. Cranial MRI revealed a low and focal contrast-enhancing nodular tumor in the left cerebellar hemisphere and upper vermis (Figure 1). The lesion appeared hypointense relative to gray matter on T1 weighted (T1w) images and moderately hyperintense on T2w images. Tumor margins were best displayed on diffusion weighted images (DWI). The suspected clinical diagnosis was metastasis from an unknown primary tumor. Microsurgical resection of the tumor was performed. Histopathological work-up revealed a highly cellular tumor consisting of small cells with scant cytoplasm and round-oval or pleomorphic, hyperchromatic cells in the cerebellum. A part of the tumor showed a nodular architecture and a desmoplastic component (Gomori staining). Some tumor cells expressed the neuronal differentiation marker synaptophysin. The diagnosis was paucinodular desmoplastic MB (WHO grade IV). Tumor cells showed nuclear YAP1 and cytoplasmic GAB1 staining, while nuclear staining for ß-catenin and staining for p53 was negative. There was no MYC- or MYCN-amplification detectable (Figure 2). The patient's condition was stable in the continuing course; however a week later he was affected by a severe pneumonia and died due to respiratory insufficiency.

**Figure 3:**
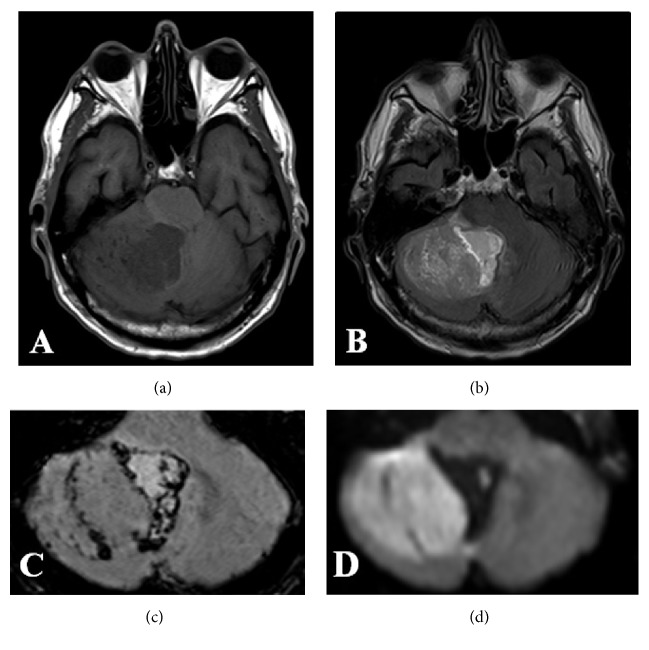
Transversal T1w image at 1.5 Tesla (a) shows a large hypointense tumor of the right cerebellar hemisphere with a hyperintense cyst on FLAIR images (b), which fills most of the fourth ventricle and causes obstructive hydrocephalus (not shown). Susceptibility weighted images (c) reveal hemosiderin deposits in the caudal tumor parts. Because of its dense cellularity, this tumor shows moderate restriction on diffusion weighted images (d).

**Figure 4:**
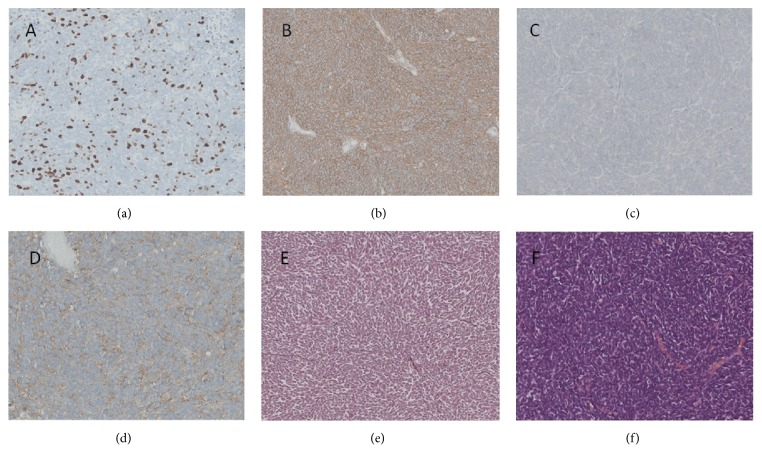
A small, round, blue cell, neuroectodermal tumor ((a), HE) without nodular architecture and without reticulin fiber network ((b), Gomori). Tumor cells showed expression of synaptophysin (c) but no expression of YAP1 and GAB1 (d). There was no nuclear expression of beta-catenin (e). Ki67-labelling reveals high proliferative activity (f).

Case 2 was a 72-year-old male who was referred to the hospital because of change of personality and loss of weight. Cranial MRI showed a large low contrast-enhancing mass in the right cerebellar hemisphere consisting of a lateral solid component and a small medial cystic. The tumor caused occlusive hydrocephalus but no surrounding edema (Figure 3). MR revealed diffusion restriction of the solid tumor part and peripheral susceptibility effects, e.g., hemosiderin deposits. Once again, the first suspected diagnosis was metastasis without presence of any neoplasm in the patient history; the second radiological diagnosis was MB. The possibility of a high-grade glioma was discussed but neglected due to its rare occurrence in the cerebellum in this age group. Prior to surgery an external ventricular drainage was inserted. Complete tumor resection was performed. Histopathological examination showed a highly cellular cerebellar tumor consisting of sheets of uniform cells with a high nuclear/cytoplasmic ratio and round to oval hyperchromatic nuclei. Many tumor cells reacted for synaptophysin. There was no evidence of a nodular or desmoplastic component in the Gomori staining. The diagnosis was that of a classical MB (WHO grade IV) (Figure 4). The tumor cells did not show staining for YAP1, GAB1, and p53 or nuclear staining for ß-catenin. Evidence of MYC- or MYCN-amplification was not found. The postoperative course was uneventful and the ventricular drainage was removed without evidence of an enlarged ventricular system. However, the patient was found dead seven days later in his room. The cause of unexpected death could not be clarified, since an autopsy was not allowed.

## 3. Discussion

Up to now there are four different molecular subtypes of MBs recognized, including activation of WNT-signaling pathway, SHH-signaling pathway, and the Non-WNT-/Non-SHH-subtype, which can be subdivided into group 3 (mostly MYC-amplified) and group 4 (often MYCN-amplified). Most of the desmoplastic MBs are of the SHH-activated subtype, whereas classic MBs are of the WNT- or the Non-WNT-/Non-SHH-subtype.

In contrast to children, in young adults the most common molecular subtypes are the SHH-activated/p53-wildtype and group 4 MB [4, 5]. Only five cases of MB occurring beyond the 70th year of life have been reported [6, 7]. All the reported cases were diagnosed as classical MB (WHO grade IV).

The two cases presented here represent also one classical MB and one additional desmoplastic MB. To our knowledge we report for the first time that there are different molecular subtypes of MB in the elderly patients that seem to be consistent with those subtypes mainly occurring in young adults.

Unfortunately the presented cases had a very unsatisfactory postoperative course, so that we cannot report any response of the patients to the planned radiotherapy in case 1 and combined radiochemotherapy in case 2.

Regarding the radiologic findings, we could not detect any differences to MB in younger ages. A moderate contrast-enhancing mass in T1-weighted sequences in the posterior fossa with surrounding brain edema and anatomical contact to the vermis and the fourth ventricle were typical findings. Histologic examination did not show any differences compared to the findings of MB occurring in younger patients.

The presented cases show that MB can occur in the elderly. Although this constellation is absolutely rare, MB should be considered in the differential diagnosis, especially when a primary tumor is not known or detected.

## References

[1] Abacioglu U., Uzel O., Sengoz M., Turkan S., Ober A. (2002). Medulloblastoma in adults: Treatment results and prognostic factors. *International Journal of Radiation Oncology • Biology • Physics*.

[2] Zhang N., Ouyang T., Kang H., Long W., Thomas B., Zhu S. (2015). Adult medulloblastoma: clinical characters, prognostic factors, outcomes and patterns of relapse. *Journal of Neuro-Oncology*.

[3] Vigneron C., Antoni D., Coca A. (2016). Adult medulloblastoma: Retrospective series of 21 patients. *Cancer Radiothérapie*.

[4] Zhao F., Ohgaki H., Xu L. (2016). Molecular subgroups of adult medulloblastoma: A long-term single-institution study. *Neuro-Oncology*.

[5] Rodriguez F. J., Eberhart C., O'Neill B. P. (2007). Histopathologic grading of adult medulloblastomas. *Cancer*.

[6] Huppmann A. R., Orenstein J. M., Jones R. V. (2009). Cerebellar medulloblastoma in the elderly. *Annals of Diagnostic Pathology*.

[7] Liang B., Feng E., Wang Q. (2016). Medulloblastoma in an elderly patient: A case report and literature review. *Molecular and Clinical Oncology*.

